# CT‐guided percutaneous transthoracic needle biopsy (PTNB): A thoracic surgeon's learning curve and experience summary

**DOI:** 10.1111/1759-7714.14793

**Published:** 2023-01-17

**Authors:** Tao Hong, Guijuan Ji, Teng Sun, Xin Gui, Tianyue Ma, Hao Zhang

**Affiliations:** ^1^ Thoracic Surgery Laboratory Xuzhou Medical University Xuzhou China; ^2^ Department of Thoracic Surgery Affiliated Hospital of Xuzhou Medical University Xuzhou China; ^3^ Department of Respiratory and Critical Care Medicine Affiliated Hospital of Xuzhou Medical University Xuzhou China

**Keywords:** CT, learning curve, lung, needle biopsy, percutaneous

## Abstract

**Background:**

Few studies have investigated the learning process of percutaneous transthoracic needle biopsy (PTNB). Here, we aimed to evaluate the number of cases required to achieve proficiency by plotting the learning curve of PTNB.

**Methods:**

Data were collected from 94 consecutive patients who underwent computed tomography–guided PTNB by a thoracic surgeon at the Affiliated Hospital of Xuzhou Medical University between May 2021 and February 2022. The data collected included patient information, relevant examination results, intraoperative parameters, postoperative complications, and diagnostic results.

**Results:**

The inflection points of the cumulative sum curve were around cases 13 and 24, according to which three phases were identified, including phase I, phase II, and phase III. A significant downtrend was observed regarding operative time (phase I, 26.53 ± 9.13 min vs. phase III, 18.42 ± 4.29 min, *p* < 0.05), rate of false‐negative (phase I, 15.4% vs. phase III, 5.7%; *p* < 0.05), rate of pneumothorax (phase I, 30.8% vs. phase III, 12.9%; *p* < 0.05), and rate of hemoptysis (phase I, 15.4% vs. phase III, 2.9%; *p* < 0.05).

**Conclusions:**

Thirteen cases were accumulated to lay the technical foundation, and 24 cases were required to achieve proficiency. In this study we summarize our own experience and provide specific guidance for young doctors with no experience in biopsy.

## Graphical abstract

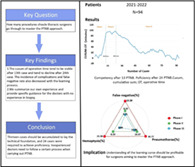

## INTRODUCTION

Lung cancer is one of the most prevalent cancers and the leading cause of cancer‐associated death worldwide.^1^ With the popularization of computed tomography (CT) in lung cancer screening and the increase in awareness of physical examination, the detection rate of pulmonary nodules is increasing annually.^2^, ^3^ The demand for lung tumor tissue samples is increasing because of the clinical needs of the pathological results of lung tumors and the importance of information on genetic mutation status in targeted drug selection for lung cancer.^4^, ^5^

Surgery is the main diagnosis and treatment of lung cancer. However, surgery cannot handle all situations. Typically, indeterminate pulmonary nodules have low‐to‐intermediate probability of malignancy.^6^, ^7^, ^8^ In particular, surgical diagnosis and treatment have disadvantages in common pulmonary nodules whose nature is difficult to determine clinically, as well as in pulmonary nodules in which pathological results are necessary to determine the treatment method.^9^, ^10^, ^11^ In contrast, CT‐guided percutaneous transthoracic needle biopsy (PTNB) has the advantages of safety, effectiveness, accuracy, and convenience and is widely used in clinical practice.^12^, ^13^, ^14^

According to the *BTS guidelines for radiologically guided lung biopsy*,^15^ percutaneous needle biopsy plays a crucial role in the diagnosis, staging, and treatment planning for tumors in the lungs, thoracic wall, hilum, and mediastinum.^16^ From its earliest use in pathological diagnosis to the classification of tissue subtypes and genetic diagnosis, the clinical need for percutaneous needle biopsy is increasing.^17^ It has become especially important to improve Chinese professionals' and technicians' understanding of PTNB, as well as to standardize operating procedures and strengthen perioperative management.^18^

However, guidelines that specify the number of procedures required and the method for performance assessments for CT‐guided PTNB are currently lacking, and the related research is rarely reported. Rohee Park et al^19^ studied the learning curve for PTNB of thoracic imaging fellows, while in most hospitals, PTNB is operated by thoracic surgeons. Due to the accumulation of different knowledge and experience, we believe that the learning curve of thoracic surgeons should be different from that of thoracic imaging fellows. Thus, the number of procedures required to reach a plateau for CT‐guided localization of small pulmonary nodules by thoracic surgeons is still unclear.

Therefore, we retrospectively collected the cases of CT‐guided biopsy performed by a thoracic surgeon in our center from scratch to explore the learning curve of this operation, explore the number of cases needed to master the skills of CT‐guided puncture biopsy, and provide experience and guidance to thoracic surgeons to learn this skill.

## METHODS

### Patients

In this study, we reviewed consecutive 94 patients who underwent CT‐guided PTNB by a thoracic surgeon at the Affiliated Hospital of Xuzhou Medical University between May 2021 and February 2022. The operator in this study was a young doctor who has just passed the examination of attending doctor after completing standardized training with no previous experience in PTNB. All patients had biopsy needs and indications.

### Data collection

The patients' demographics (age and sex), procedure date, target lesion characteristics (lesion size, location, type, and depth from the pleura), patient position during the procedure, the presence of emphysema along the needle pathway, procedure time, and pathological results were recorded. The presence of pneumothorax was assessed on postprocedural CT images or follow‐up radiographs acquired within 7 days of the procedure. Pneumothorax requiring chest tube insertion and the occurrence of hemoptysis were recorded. The operation time was defined as the interval time between the first CT scan and the last CT scan of the procedure. Pneumothorax was defined as no gas in the chest during the first CT scan, but gas appeared during the last CT scan. According to the lung compression volume, it could be divided into small pneumothorax of less than 30%, medium pneumothorax of 30%–70%, and large pneumothorax of more than 70%. The diagnosis of emphysema was based on the results of the chest CT report before operation.

### Biopsy procedure

The operator initially observed several procedures that were performed by an experienced faculty thoracic surgeon. Then, the operator performed CT‐guided PTNBs under the supervision of an experienced thoracic surgeon. Before each procedure, the operator reviewed available imaging examinations and planned the most effective needle pathway that would avoid bulla, emphysema, large proximal pulmonary vessels, airways, and fissures. A standard coaxial technique was performed using a 17‐gauge coaxial introducer and an 18‐gauge cutting needle (Pro‐Mag 2.2, Manan Medical Products). Interval CT scans were acquired to evaluate the pathway of the coaxial introducer during the procedure. After a coaxial introducer was properly positioned at the target lesion, the biopsy was performed using an 18‐gauge cutting needle. A procedure CT scan was performed to detect immediate complications. The presence of pneumothorax was assessed on postprocedural CT images or follow‐up radiographs acquired within 7 days of the procedure. Pneumothorax requiring chest tube insertion and the occurrence of hemoptysis were recorded.

### Assessment of diagnostic performance

The pathological diagnosis of the malignant pulmonary nodules could be obtained in two ways, namely surgical resection and lung biopsy. Similarly, the pathological diagnosis of benign SPNs could also be made via three ways: (a) surgical resection; (b) if the lung biopsy results indicated specific benign results (benign tumors, organizing pneumonia, or tuberculosis); and (c) the lesions narrowed during later follow‐up, and they could be accepted as the final diagnosis. All pathological results were confirmed by more than two pathologists.

### Cumulative sum (CUSUM) analysis

Learning curves provide a graphical representation between learning effort (i.e., number of procedures performed) and learning outcomes.^20^, ^21^ CUSUM analysis was applied to the whole series to investigate the number of operated cases after which a stable learning curve, represented by improved operative time, was achieved.^22^ The CUSUM analysis of operative time was assessed as P (Xi−X0), where Xi was the individual operative time for each case, and X0 was the reference level that was set as the mean operative time for the whole series.^23^

The CUSUM score of the occurrence of pneumothorax was recorded from p0, indicating an acceptable failure rate (the level of inherent error if the procedure is performed correctly), and p1, indicating an unacceptable failure rate. The CUSUM score was generated at the beginning as 0. After each case of success, an amount “s” was subtracted from the previous CUSUM score, and an amount 1‐s was added to the previous CUSUM score for each case of failure. A successful outcome led to downward movement of the CUSUM score, whereas a failed outcome led to upward movement of the CUSUM score.

When the CUSUM line crossed the lower decision threshold (H0), learning was considered to have been achieved. If the CUSUM crossed the upper decision threshold (H1), learning was considered to have failed. If the CUSUM line remained between H0 and H1, the presence of learning was considered inconclusive. These limits were calculated based on the risk for type I and type II errors. A type I error (α) was the probability of failing to cross the acceptable limit when the true failure rate was within the acceptable range, whereas a type II error (*β*) was the probability of downward crossing of the acceptable failure rate limit when the true failure rate was not in the acceptable range. In this study, type I and type II error levels of 0.10 were used for all analyses. The p0 and p1 in this analysis were determined to be 0.25 and 0.45, respectively, for pneumothorax occurrence. From among the wide range of reported pneumothorax rates of between 0.10 and 0.29, an acceptable pneumothorax occurrence rate of 0.25 was set,^24^, ^25^ with an unacceptable pneumothorax occurrence rate of 0.45 according to the suggested quality improvement threshold.^26^ Other values adopted in this study are summarized in Table 1.

**TABLE 1:** Demographic and clinical characteristics of patients

Parameters	Total	Phase I (*n* = 13)	Phase II (*n* = 11)	Phase III (*n* = 70)	*p*‐value (phase I vs. II)	*p*‐value (phase II vs. III)	*p*‐value (phase I vs. III)
Age, years	62.47 ± 10.12.59	61.46 ± 10.65	59.91 ± 13.33	63.07 ± 12.83	0.754	0.555	0.792
Sex, *n*(%)					0.095	0.042	0.748
Male	63 (67.0%)	10 (76.9%)	4 (63.6%)	49 (70.0%)			
Female	31 (33.0%)	3 (23.1%)	7 (36.4%)	21 (30.0%)			
Smoking history, *n*(%)	39 (41.5%)	4 (30.8%)	4 (36.4%)	31 (44.3%)	0.957	0.749	0.542
Emphysema present	11 (41.5%)	3 (23.1%)	2 (18.2%)	6 (8.6%)	0.773	0.653	0.290
Pulmonary tuberculosis	0	0	0	0			
Lesion size, mm	51.24 ± 24.19	62.83 ± 30.26	44.54 ± 16.22	49.96 ± 23.58	0.089	0.472	0.113
Lesion location					0.318	0.077	0.747
LUL	25 (26.6%)	2 (15.4%)	0 (0.0%)	23 (32.8%)			
LLL	16 (17.0%)	2 (15.4%)	3 (27.3%)	11 (15.7%)			
RUL	21 (22.3%)	3 (23.1%)	5 (45.4%)	13 (18.6%)			
RML	8 (8.5%)	2 (15.4%)	0 (0.0%)	6 (8.6%)			
RLL	24 (25.5%)	4 (30.8%)	3 (27.3%)	17 (24.3%)			
Nodule type							
Ground‐glass	2 (2.1%)	0 (0.0%)	0 (0.0%)	2 (2.9%)			
Solid	92 (97.9%)	13 (100.%)	11 (100.%)	68 (97.1%)			

*Note*: Data are presented as mean ± SD, or number (percentage).

Abbreviations: LLL, left lower lobe; LUL, left upper lobe; RLL, right lower lobe; RML, right middle lobe; RUL, right upper lobe.

### Statistical analysis

Based on the inflection points, we separated the curve of operative time into three distinct phases: Phase I, phase II, and phase III. A subgroup analysis was conducted by comparing the perioperative outcomes of the three periods to assess a potential learning curve effect. All statistical analyses were conducted with SPSS, version 22 (IMB Corp, Inc.). Normally distributed continuous variables are presented as the mean ± standard deviation and were compared by independent‐sample *t*‐test. One‐way analysis of variance was used to analyze the data among the three groups. Categorical data were analyzed using the chi‐square test, Pearson's test, or Fisher's exact test. A *p*‐value <0.05 was considered statistically significant.

## RESULTS

### Patient characteristics

A total of 94 PTNB cases, which were performed continuously by an inexperienced thoracic surgeon, were collected between May 2021 and February 2022. These patients consisted of 63 (67.0%) male and 31 (33.0%) female patients with a mean age of 62.47 years old. More detailed patient characteristics are presented in Table 1.

### Learning curves

**FIGURE 1:**
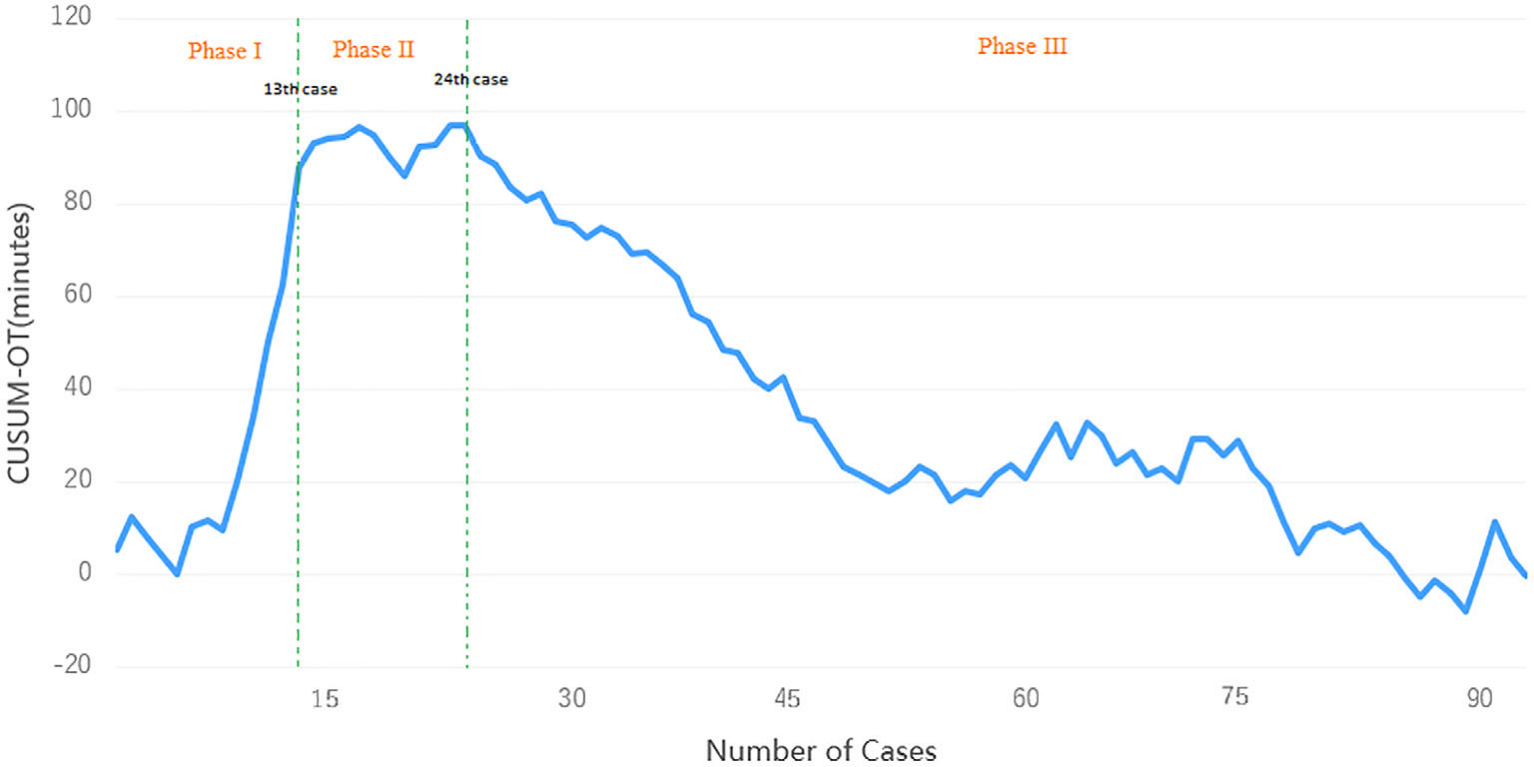
Learning curve of operative time. Competency after 13 percutaneous transthoracic needle biopsies (PTNB). Proficiency after 24 PTNB. Cusum, cumulative sum; OT, operative time.

**FIGURE 2:**
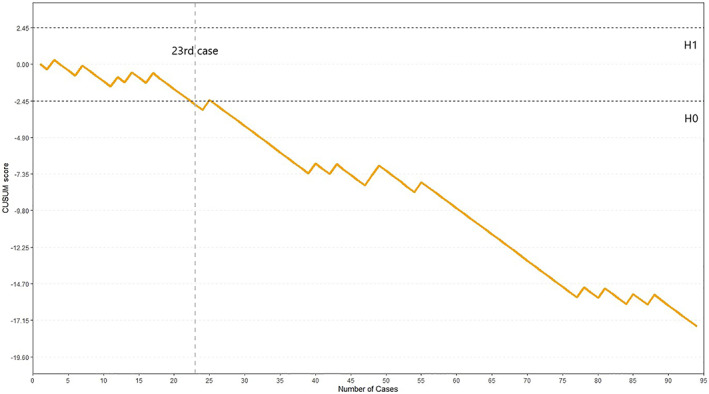
Learning curve of pneumothorax. Proficiency after 23 percutaneous transthoracic needle biopsies (PTNB). Cusum, cumulative sum.

As illustrated in Figure 1, two inflection points fell at the 13th case and the 24th case. Phase I (*n* = 13, 1–13 cases) represented the initial experience with the technique; phase II (*n* = 11, 14–24 cases) represented the further improvement of surgical skills; and phase III (*n* = 70, 25–94 cases) indicated that technical proficiency was achieved. A visual inspection of the CUSUM plot showed that the cumulative operative time increased in phase I, hit a plateau in phase II, and decreased in phase III (Figure 1). According to the learning curve of pneumothorax rate (Figure 2), the operator achieved this after the 23rd case. This result is roughly the same as CUSUM‐ot.

### Diagnostic accuracy

All 94 PTNBs were technically successful. The mean procedure time was 19.8 min. A total of 74 lesions were confirmed as malignant, and 12 lesions were confirmed as benign. The overall sensitivity, specificity, and accuracy for the diagnosis of malignancy were 91.4% (74 of 81), 100% (13 of 13), and 92.6% (87 of 94), respectively (Table 3).

### Pneumothorax occurrence

The incidence of pneumothorax in this study was 16.0%, and none of the patients required chest tube insertion. All patients with pneumothorax remained in bed after puncture, and their symptoms were relieved after oxygen inhalation. CT pneumothorax was improved before discharge. The incidence of pneumothorax in the first stage was 30.88%, in the second stage was 18.18%, and in the third stage was 12.68% (Table 2).

**TABLE 2:** Perioperative parameters and complications

Parameters	Total	Phase I (*n* = 13)	Phase II (*n* = 11)	Phase III (*n* = 70)	*p*‐value (phase I vs. II)	*p*‐value (phase II vs. III)	*p*‐value (phase I vs. III)
Patient position					0.979	0.179	0.185
Supine	37 (39.4%)	4 (30.8%)	4 (36.4%)	30 (42.3%)			
Prone	47 (50.0%)	6 (46.1%)	5 (45.4%)	36 (50.7%)			
Lateral	10 (10.6%)	3 (23.1%)	2 (18.2%)	5 (7.0%)			
Emphysema					0.649	0.806	0.482
Absent	73 (77.7%)	9 (69.2%)	9 (81.8%)	55 (78.6%)			
Present	21 (22.3%)	4 (30.8%)	2 (18.2%)	15 (21.4%)			
Operation time	19.79 ± 5.81	26.53 ± 9.13	20.63 ± 3.5	18.4 ± 4.31	0.560	0.806	<0.001
False‐negative, *n*(%)	7 (7.4%)	2 (15.4%)	1 (9.1%)	4 (5.7%)	0.183	0.783	0.063
Pneumothorax, *n* (%)					0.310	0.533	0.003
Yes	15 (16.0%)	4 (30.8%)	2 (18.2%)	9 (12.9%)			
No	79 (84.0%))	9 (69.2%)	9 (81.8%)	61 (87.1%)			
Hemoptysis, *n* (%)					0.183	0.213	0.005
Yes	5 (5.3%)	2 (15.4%)	1 (9.1%)	2 (2.9%)			
No	89 (94.7%)	11 (84.6%)	10 (90.9%)	68 (97.1%)			
Depth of needle, cm	5.61 ± 1.62	5.55 ± 1.46	6.91 ± 1.81	5.44 ± 1.54	0.054	0.006	0.825
Number of CT scans					0.700	0.360	0.274
<5	3 (3.2%)	1 (7.7%)	0 (0.0%)	2 (2.9%)			
5–8	45 (47.9%)	3 (23.1%)	6 (54.5%)	36 (51.4%)			
9–12	34 (36.2%)	7 (53.8%)	1 (9.1%)	26 (37.1%)			
>12	12 (12.1%)	2 (15.4%)	4 (36.4%)	6 (8.6%)			

*Note*: Data are presented as mean ± SD, or number (percentage).

**TABLE 3:** Accuracy of computed tomography (CT)‐guided percutaneous transthoracic needle biopsy (PTNB) with 18‐gauge cutting needles

Parameters	Total	Phase I (*n* = 13)	Phase II (*n* = 11)	Phase III (*n* = 70)
True‐positive	74 (78.7%)	9 (69.2%)	9 (81.9%)	56 (80.0%)
True‐negative	13 (13.9%)	2 (15.4%)	1 (9.1%)	10 (14.3%)
False‐positive	0 (0.0%)	0 (0.0%)	0 (0.0%)	0 (0.0%)
False‐negative	7 (7.4%)	2 (15.4%)	1 (9.1%)	4 (5.7%)

*Note*: Data are presented as number (percentage).

### Hemoptysis occurrence

The incidence of hemoptysis in this study was 5.3%, with no occurrence of massive hemoptysis. The symptoms of all patients with hemoptysis disappeared after symptomatic treatment. The incidence of hemoptysis in the first stage was 15.4%, in the second stage was 9.1%, and in the third stage was 2.9% (Table 2).

## DISCUSSION

CT‐guided PTNB has been proven to be a safe and feasible diagnosis method for lung cancer. However, the accuracy and safety of PTNB are greatly affected by the operator's proficiency. In this study, we investigated 98 cases of PTNB conducted by a single surgeon at the Affiliated Hospital of Xuzhou Medical University to evaluate our learning curve using CUSUM analysis.

Although previous studies have demonstrated the safety and feasibility of PTNB despite its technical difficulties, few have explored the learning phase of this complex procedure. The current analysis is a valuable supplement for several reasons. First, we highlight the time course and caseload required to achieve proficiency by rendering the learning curve, which is profitable for future mentorship. Additionally, we investigated several measures that could improve PTNB outcomes during the learning process.

**FIGURE 3:**
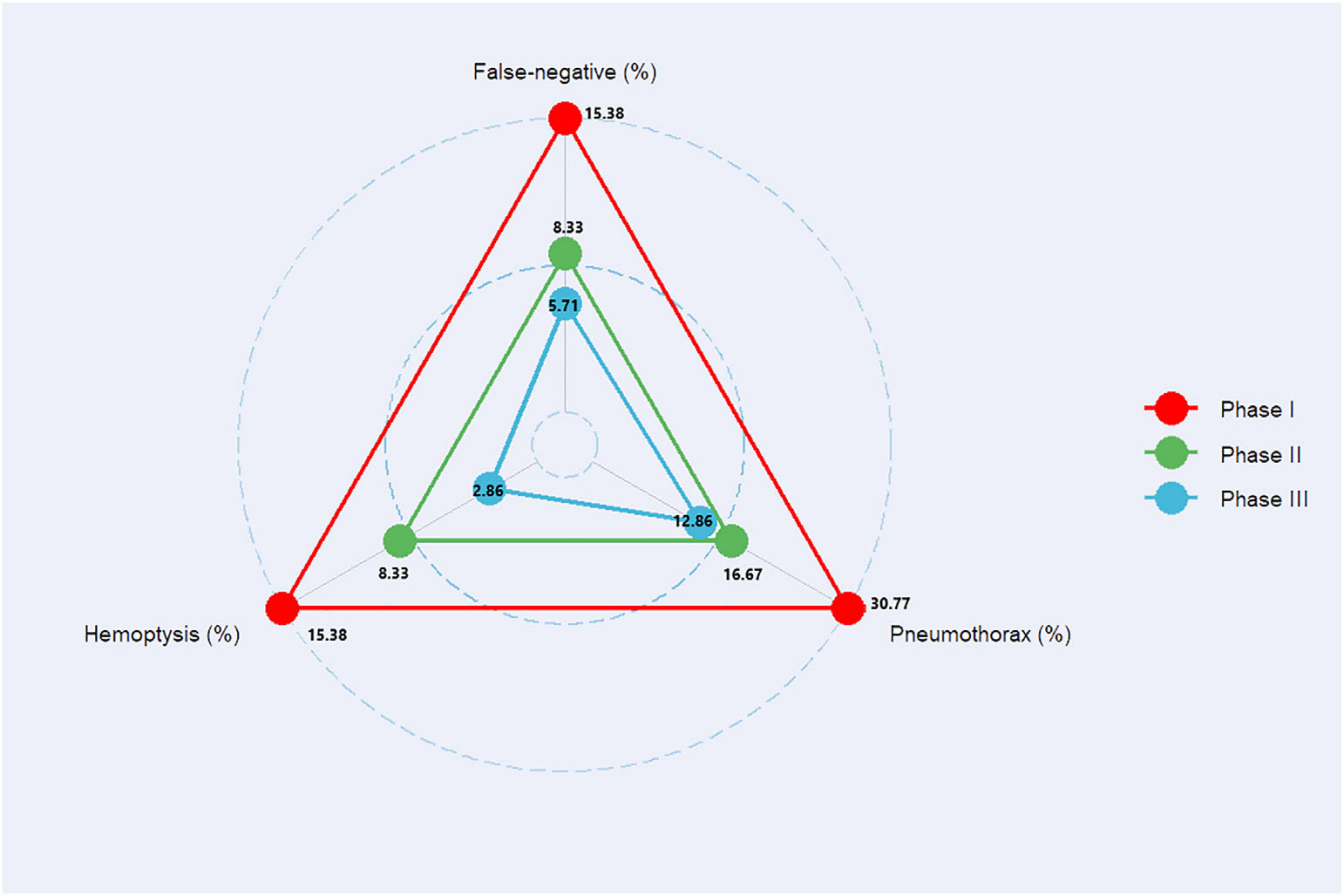
Perioperative outcomes. Perioperative outcomes including false negative rate, pneumothorax, and hemorrhage improved as the learning curve developed.

In our study, CT‐guided PTNB showed an overall accuracy of 92.6% (87 of 94). The operators reached the platform stage after completing the practical operation of 13 cases and reached the proficiency stage after 24 cases. The accuracy rates of learning, platform, and proficiency stages were 84.6, 90.1, and 94.3%, respectively (Figure 3). The number of cases required in the three stages of this study was consistent with the experience of many thoracic surgeons with mature puncture biopsy experience in our center.

In practice, researchers have found that the location of lesions impacts the accuracy and safety of diagnosis. Lesions in the lower lobe were an independent risk factor in the study by Lee et al.^8^ and may be explained by respiratory motion, which is greatest in the lower lobe. Therefore, CT scan and needle insertion at the end of deep inspiration or deep expiration can help to reduce the deviation caused by breathing.

In this study, the pneumothorax rate was 16.0% (15 of 94), and the incidence of chest drainage catheter placement in patients with PTNB‐related pneumothorax was 0% (0 of 94). The incidence of pneumothorax in the learning period was 30.8%, in the platform period was 18.2%, and in the proficiency period was 12.9% (Figure 3). These rates are within the previously reported ranges for pneumothorax rate (2.4% to 60%, average: 20%) and incidence of chest drainage catheter placement (range, 5%–18%) after PTNB.^27^ Significant risk factors for pneumothorax are emphysema along the needle pathway, two or more pleural passages, lesions in the lower lobe, and male sex. Cox et al.^28^ also reported emphysema along the needle pathway as a risk factor for the occurrence of pneumothorax, which can be explained by the fact that the disruption of dilated air space can increase airway pressure and prevent rapid sealing of air leak. Two or more pleural passages are considered a risk factor because more pleural passages lead to more visceral pleural injuries, which appear to be associated with the occurrence of pneumothorax. Concerning lesions in the lower lobes, it is possible that greater respiratory motion in the lower lobes may require more redirections of the needle to reach the lesion and cause the needle to further damage the lung parenchyma. Male sex may be associated with a greater forced vital capacity,^29^ which has been shown to be significantly related to a greater likelihood of the occurrence of pneumothorax in the study by Hiraki et al.^30^

In the current study, the hemoptysis rate of 5.3% (5 of 94) was slightly higher than the reported incidence (range, 1.25%–7%)^27^ of hemoptysis in CT‐guided biopsy. Most cases of hemoptysis occur in the early stage of learning (Figure 3), and all disappeared after symptomatic treatment. In practice, researchers have found that deep lesions have a greater risk of bleeding during the operation, as reported by Yeow et al. This can be explained by the fact that a long needle path and tissue sampling of centrally located lesions have a higher chance of vascular injuries both along the needle course and in the central pulmonary vasculature. Summarized by the researcher's experience, choosing an appropriate route to shorten the depth of the needle and avoid blood vessels can effectively reduce the risk of bleeding. The appropriate puncture position and the shortest needle route should be chosen according to the location of the tumor. Meanwhile, when the shortest needle route passes through a large blood vessel, another needle route should be chosen to avoid the blood vessel.

According to the learning curve for PTNB of thoracic imaging fellows studied by Rohee Park et al, at least 37 and 52 procedures are required to achieve acceptable diagnostic accuracy and false‐negative rates, respectively. Not all operators achieved acceptable complication rates. Compared with ours, their acceptable standard is lower (90%) and the number of cases required is higher. The reason may be the object of our study which is the thoracic surgeon, who has stronger hands‐on ability and greater fundamental knowledge of surgical procedures. This makes the learning process of PTNB easier for thoracic surgeons than for thoracic imaging fellows, and the learning and platform periods will also be shortened. In other words, the experience accumulated by thoracic surgeons in other operations can provide a definitive basis for learning this operation.

In addition, we note that not all operators achieved acceptable complication rates in their study. It may be because thoracic imaging fellows had no experience in dealing with relevant dangerous situations. They might have been overcautious during surgery, and thus more likely to make mistakes. Thoracic surgeons have encountered many emergencies that are more urgent and dangerous than puncture complications in clinic and surgery. Therefore, thoracic surgeons have more experience and confidence in the treatment of puncture complications, which can lead to safer and more accurate results.

**FIGURE 4:**
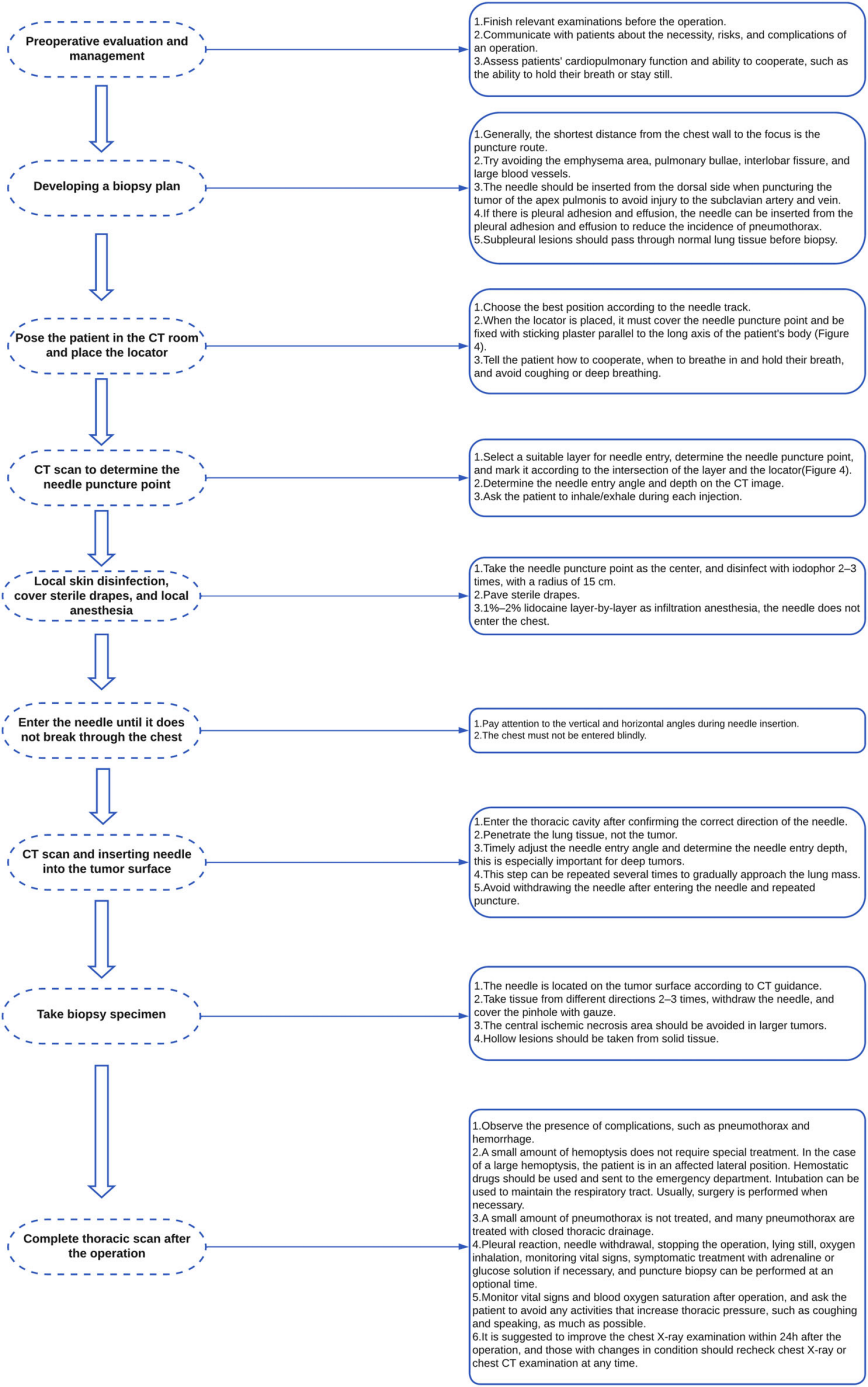
Determination of the needle entry point.

**FIGURE 5:**
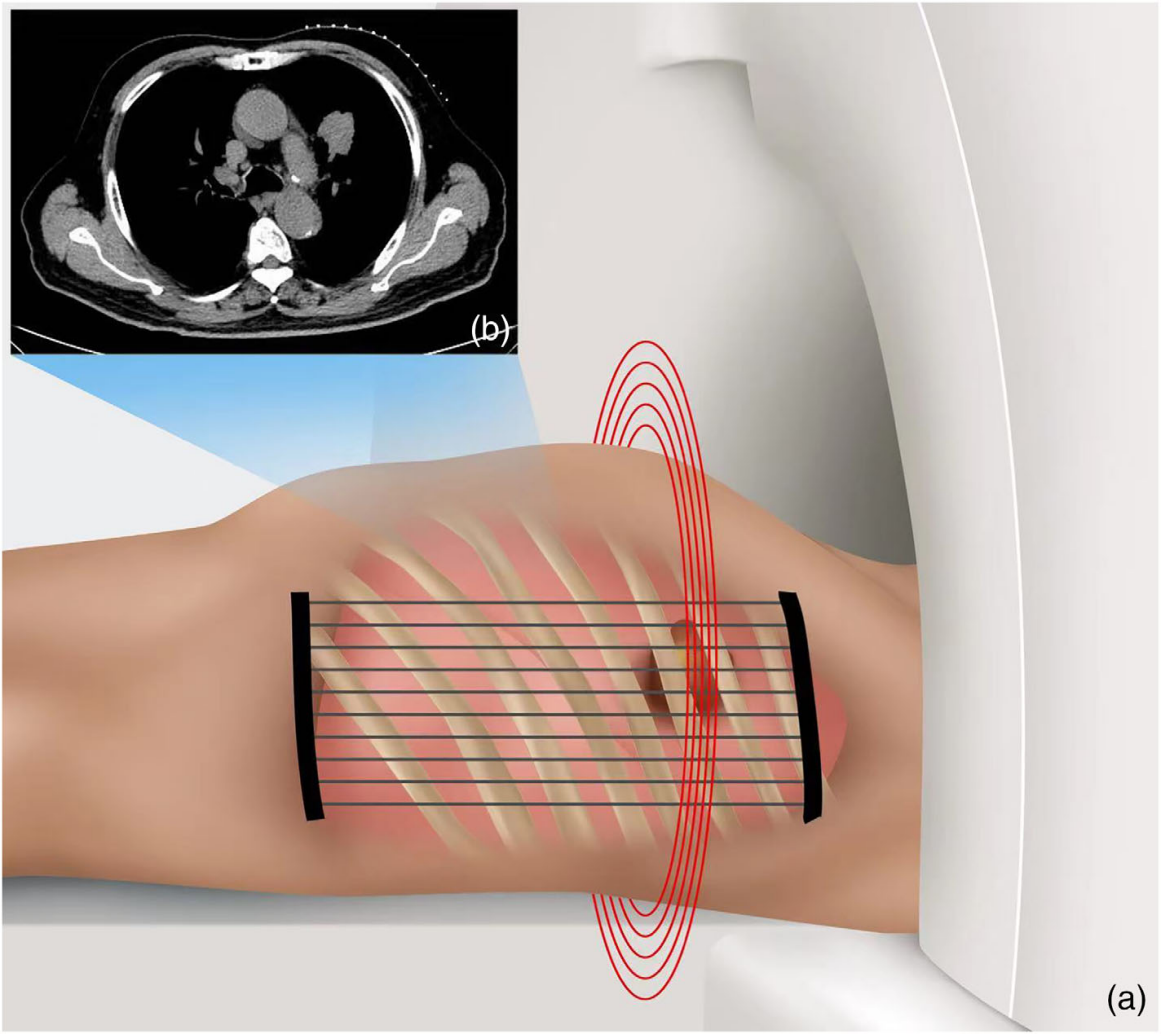
Determination of the needle entry point. (a) Place the locator parallel to the long axis of the patient's body. (b) Select a suitable layer for needle entry.

Researchers encounter many specific problems in practice, including how to puncture the tumor on the dorsal side of the lung covered by the scapula, and often cannot find answers from the guidelines and relevant literature. The operator found that the scapular moves forward to make a way for the needle when the patien is in the prone position with arms folded across the chest. To provide more specific guidance to young doctors, we summarize our own experience and developed the specific steps and precautions of PTNB along with the guidelines (Figures 4, 5).

Our study has several limitations. First, our study had a retrospective design in which selection bias is inevitable. Second, the use of data from only a single surgeon limits the introduction of our results to a general population of learners. Third, there is no definite evidence to demonstrate whether different nursing teams, radiologists, and fellows for each procedure would impact the learning curve. For this study, we focused on the evaluation of accuracy and safety. Multicenter trials with a larger sample size must be conducted in the future to verify the safety profile.

## AUTHOR CONTRIBUTIONS

Tao Hong: Propose research topics; Design research plan; Implement the research process; Collect and sort out data; Statistical analysis data; Writing papers. Guijuan Ji: Implement the research process; Collect and sort out data; Statistical analysis data; Writing papers. Teng Sun: Design research plan; Implement the research process; Implement the research process; Collect and sort out data. Xin Gui: Implement the research process; Collect and sort out data; Statistical analysis data; Drawing and tabulation. Tianyue Ma: Implement the research process; Collect and sort out data; Statistical analysis data; Drawing and tabulation. Hao Zhang: Propose research topics; Design research plan; Implement the research process; Review and modify; Financial support.

## CONFLICT OF INTEREST

The authors declare that they have no conflict of interest.
